# Bioenergetics of cancer cells: insights into the Warburg effect and regulation of ATP synthase

**DOI:** 10.1186/s10020-025-01378-0

**Published:** 2025-10-13

**Authors:** Valentina Del Dotto, Silvia Grillini, Riccardo Righetti, Martina Grandi, Valentina Giorgio, Giancarlo Solaini, Alessandra Baracca

**Affiliations:** Department of Biomedical and Neuromotor Sciences, University of Bologna, Via Irnerio 48, Bologna, 40126 Italy

**Keywords:** Aerobic glycolysis, Cancer, Bioenergetics, Mitochondria, IF_1_, ATP synthase, ROS

## Abstract

The study reported here offers new insights into the metabolic changes associated with the Warburg effect (i.e. aerobic glycolysis) in cancer cells and into the possible role of IF_1_, the endogenous inhibitor of ATP synthase that preserves cellular energy when it works in reverse, hydrolyzing ATP. We investigated biochemical and main bioenergetic parameters in cell lines derived from three human tumors: osteosarcoma (143B), colon carcinoma (HCT116), and cervix carcinoma (HeLa). The combination analysis of cellular glucose consumption, lactate production, ATP-linked respiration rate, ATP level, cell culture medium acidification rate, and ROS level demonstrates that aerobic glycolysis is differently expressed by the three different types of tumor cells, although all cell types exhibited a Warburg phenotype. The superoxide anion level was found to be lower in HCT116 cells, which showed the highest ratio between oxidative phosphorylation and glycolysis rates, while ROS level was similar in all cells examined, suggesting that mitochondria in HCT116 are very efficient in both energy production and limiting their oxidative stress. Additionally, IF_1_ KD cells of all kinds of tumor showed higher level of ROS compared to their related IF_1_-expressing cells. Most of the results reported here clearly demonstrate that aerobic glycolysis is completely independent on both the level of IF_1_ and the IF_1_/ATP synthase ratio, excluding the contribution of an IF_1_-dependent mechanism in the metabolic shift of cancer cells towards glycolysis. Indeed, the study provides a detailed analysis of the bioenergetics of tumor cells exhibiting very different IF_1_/ATP synthase ratios and shows that IF_1_ KD cells of all tumor types had a higher level of ROS than their related IF_1_-expressing cells. Overall, the comprehensive picture of tumor cell bioenergetics would facilitate the identification of appropriate drugs for targeted tumor treatments, such as ATP synthase-IF_1_ immunotherapy that would strongly limit cellular resistance to severe hypoxia or anoxia, where IF_1_ plays an effective critical role.

**Supplementary Information:**

The online version contains supplementary material available at 10.1186/s10020-025-01378-0.

## Introduction

The Warburg effect (also called “aerobic glycolysis”) was originally proposed in 1927 by Warburg et al. who observed a high rate of glucose consumption with persistent lactate production (fermentation) in mammalian cancer cells, even in the presence of sufficient oxygen to support oxidative phosphorylation (OXPHOS) (Warburg et al. 1927). Subsequently, this observation was further investigated and this aerobic phenomenon was attributed to dysfunctions of mitochondrial respiration (Warburg 1956). More recently, the particularly high glycolytic flux in cancer cells has been shown to be due to mis-expression and/or mutation of tumor suppressor genes (loss-of-function effects) and oncogenes (gain-of-function effects) such as MYC and K-Ras (Singh et al. 2025; Sorolla et al. 2020; Baracca et al. 2010), and overexpression of the pro-oncogenic factor HIF (Semenza 2003; Solaini et al. 2011). All the latter factors are able to induce the expression of glucose transporters, glycolytic enzymes and isoforms of critical enzymes that control glycolytic flux. For instance, HIF-1 shifts the balance of energy metabolism toward glycolysis by coordinating two different actions, increasing the levels of both glucose transporters and glycolytic enzymes and activating the transcription of both *PDK1* and *LDHA* genes (Wicks and Semenza 2022). *LDHA* encodes lactate dehydrogenase, which converts pyruvate to lactate, and PDK1 is a kinase that phosphorylates and inactivates pyruvate dehydrogenase slowing down the feeding rate of Acetyl-Coenzyme A to the TCA cycle, that in turn decreases its coupled OXPHOS rate (Yeung et al. 2008; Stine et al. 2015; Li et al. 2016). The metabolic reprogramming is functional to cancer cells to support their unrestricted growth, which has been recognized as one of the hallmarks of cancer (Hanahan and Weinberg 2011). Indeed, rewiring allows cancer cells to both meet the large demand for glycolytic intermediates and reducing equivalents (NADPH) to produce mass, and to rapidly supply ATP to provide the high anabolism necessary for the high rate of cell proliferation (DeBerardinis et al. 2008; Pavlova and Thompson 2016; Martins Pinto et al. 2023). Despite numerous research evidence, including those mentioned above, aimed at explaining at the molecular level the Warburg effect in cancer, a mitochondrial biochemical mechanism has been proposed (Sánchez-Cenizo et al. 2010; García-Bermúdez et al. 2015). Since the F_1_F_o_-ATPase (ATP synthase) inhibitor protein, IF_1_, has been found overexpressed in many types of human tumors, the authors proposed IF_1_ as a factor that determines, or contributes to, aerobic glycolysis by inhibiting OXPHOS, mimicking the action of oligomycin, the well-known and selective inhibitor of ATP synthase. However, the tight control of ADP phosphorylation by the mitochondrial proton motive force (Δµ_H_+) whose membrane potential, *ΔΨ*_m_, is the major component, prevents the small endogenous protein IF_1_ from binding to and inhibiting ATP synthase under conditions of physiological or higher oxygen tension (Schwerzmann and Pedersen 1981; Klein and Vignais 1983; Lippe et al. 1988; Rouslin and Broge 1993). This should exclude that IF_1_ plays a significant role in mediating the switch of cancer cells from OXPHOS-dependent metabolism to aerobic glycolysis [see (Solaini et al. 2021) for a recent review]. According to the literature, IF_1_ binds and inhibits the ATP synthase of cellular and animal models only when *ΔΨ*_m_ is collapsed and the enzyme works in reverse, hydrolyzing ATP (Rouslin and Broge 1993; Bosetti et al. 2000; Campanella et al. 2008; Sgarbi et al. 2018a). Furthermore, research groups that have worked extensively on purified ATP synthase, isolated mitochondria, and sub-mitochondrial particles have never observed that IF_1_ can bind to and inhibit the enzyme when it synthesizes ATP, even when IF_1_/ATP synthase ratio is extremely high (Kobayashi et al. 2023; Carroll et al. 2024). Nevertheless, the role of IF_1_ as an inducer of the metabolic rewiring in cancer cells, by inhibiting the ATP synthase working physiologically, was subsequently reported in some other studies and was still hypothesized to be at the basis of metabolic reprogramming (Hardonnière et al. 2017; Zhou et al. 2022; Nuevo-Tapioles et al. 2020), but no direct molecular evidence of binding or interaction of IF_1_-ATP synthase as a promoter of aerobic glycolysis in cancer cells has ever been reported.

The present work was designed, first, to provide additional data to better define the bioenergetics associated with the rewiring of cancer cell metabolism and, second, to investigate whether IF_1_ plays a role in promoting the Warburg phenotype and increasing cellular reactive oxygen species (ROS), which has been proposed to be a consequence of reduced OXPHOS rate caused by the inhibition of the ATP synthesizing activity of F_1_F_o_-ATPase by IF_1_.

## Materials and methods

### Chemicals

Bovine serum albumin (A7030), digitonin (D5628), dibutyryl cyclic-AMP sodium salt (db-cAMP) (D0627), Dulbecco’s Modified Eagle Medium (DMEM) (D5030), DMEM Sigma D5030, Glucose (G7021), Glutamine (G3126), H-89 dihydrochloride hydrate (H89) (B1427), phenylmethylsulfonyl fluoride (PMSF) (P7626), Protease inhibitors (P8340), Pyruvate (P2256), sodium deoxycholate (D6750), Triton X-100 (X-100), Iodoacetamide (i6125), Oligomycin A (75351), Malonic acid (M4795), L-Malic acid (M1000), L-Glutamic acid (G1251), Sodium succinate dibasic hexahydrate (S5047), Dimethyl sulfoxide (D4540), Antimycin A from Streptomyces sp. (A8674), Rotenone (R8875), Carbonylcyanide 4-(trifluoromethoxy) phenylhydrazone (FCCP) (C2920), Oligomycin A (75351), Adenosine 5′-diphosphate sodium salt (A2754), Immobilon Forte Western HRP Substrate (WBLUF0500), β-actin (A5441) and goat anti-rabbit IgG (1706515) antibodies were all purchased from Sigma-Aldrich (Merk) (St. Luis, MO, USA). Horseradish peroxidase-conjugated secondary antibody goat anti-mouse IgG (G21040), MitoSOX™ (M36008), CellROX™ Orange Reagent (C10443) and Pierce BCA Protein Assay Kit (23225) were from Thermo Fisher Scientific (Waltham, MA, USA). ATP Bioluminescence Assay Kit LCS II (ref 1169969591) was from Roche (Basel, Switzerland). COXIV (11242-1-AP) antibody was from Proteintech (Rosemont, IL, USA). ATP5H (ab110275), NDUFS1 (ab157221), UQCRC2 (ab14745), SDHA (ab14715) and IF_1_ (ab110277) antibodies were from Abcam (Cambridge, UK).

### Cell culture

Human osteosarcoma (143B), human colon carcinoma (HCT116), human cervix adenocarcinoma (HeLa), and human embryonic kidney (HEK293) cell lines were maintained at 37 °C with 5% CO_2_ in DMEM high glucose containing 10% Fetal Bovine Serum (FBS). IF_1_-silenced (knock-down, IF_1_ KD) derived stable clones were previously reported (Barbato et al. 2015; Sgarbi et al. 2018a, 2024; Righetti et al. 2023). All the experiments were performed by seeding cells in complete medium containing 25 mM glucose, 4 mM glutamine and 1 mM pyruvate.

### Glucose consumption and lactate release

Parental and IF_1_-silenced clones were seeded in complete DMEM and, the day after, the medium was replaced with fresh medium. Glucose consumption and lactate release were determined in cells grown for 24 h either under basal condition or in the presence of 0.2 µM oligomycin, using the Glucose (Glu) Colorimetric Assay Kit (GOD-POD method) and L-Lactic Acid (LA) Colorimetric Assay Kit (Elabscience, USA), following the manufacturer’s instructions. Data normalized to the number of cells are expressed as µmol/10^6^ cells.

### Cellular ATP content detection

Parental and IF_1_-silenced clones were seeded in complete DMEM and cultured for 48 h. When required, cells were incubated with either 0.2 µM oligomycin or 0.7 mM iodoacetamide for 30 min. The ATP content of cells was assayed by a bioluminescence method using a luciferin-luciferase system (ATP bioluminescent assay kit CLS II) as previously described (Sgarbi et al. 2009). The amount of ATP measured was normalized to the protein content and expressed as nmol/mg protein.

### Oxygen consumption rate and extracellular acidification rate

The oxygen consumption rate (OCR) in adherent cells was measured using the XF96 Extracellular Flux Analyzer (Agilent technologies, Santa Clara, CA, USA), as previously reported (Bouzidi et al. 2020). Parental and IF_1_-silenced cells were seeded (12,5 × 10^3^ cells/well for 143B; 18 × 10^3^ cells/well for HCT116; 15 × 10^3^ cells/well for HeLa) in XF96 cell culture microplates and allowed to attach for 24 h. The day after, the growth medium was replaced with the Seahorse medium (DMEM Sigma D5030) supplemented with 25 mM glucose, 5 mM sodium pyruvate and 2mM glutamine, and cells were incubated at 37 °C for 30 min to allow temperature and pH equilibration. After an OCR baseline measurement, 1 µM oligomycin, 0.8 µM or 0.2 µM or 0.4 µM FCCP for 143B, HCT116 and HeLa cells, respectively, 1 µM rotenone, and 1 µM antimycin A were sequentially added to each well. Extracellular acidification rate (ECAR) was monitored simultaneously to OCR when cells were in basal condition or after 1 µM oligomycin treatment. After each experiment protein concentration in each well was quantified with the BCA protein assay and used to normalize the OCR and ECAR.

### SDS-PAGE and Western blot analysis

Cells were lysed and proteins were separated by SDS-PAGE in Bolt 4–12% Bis-Tris Plus Gels (Thermo Fisher Scientific, Life Technologies Italia, Monza, Italy), transferred onto nitrocellulose membranes and incubated with antibodies to perform semiquantitative analysis, as previously described (Sgarbi et al. 2017). Chemiluminescence detection was performed with the ECL Western Blotting Detection Reagent Kit Amershan (GE Healthcare, Merck Life Science S.r.l., Milan, Italy) using the ChemiDoc MP system equipped with ImageLab software (Bio-Rad, Bio-Rad Laboratories S.r.l., Milan, Italy) to perform the densitometric scanning and analysis of the relative protein intensity.

### Protein determination

Protein concentration was measured by the method of Lowry et al. (Lowry et al. 1951) in the presence of 0.3% (weight to volume ratio) sodium deoxycholate. Bovine serum albumin was used as standard. For OCR in adherent cell experiments, the protein quantification is performed using the Pierce BCA Protein Assay Kit.

### Citrate synthase activity

Citrate synthase activity was assayed essentially by incubating the cells with 0.02% Triton X-100 and monitoring the reaction by measuring spectrophotometrically the rate of free coenzyme A released, as previously reported (Sgarbi et al. 2018c).

### Mitochondrial ATP synthesis assay

HEK293 cells and IF_1_-silenced clones were seeded in complete DMEM and 48 h later, when required, were treated with the PKA agonist: 100 µM db-cAMP or 10 µM competitive inhibitor H89 for 12 h before processing. The oligomycin-sensitive ATP synthase activity was determined in permeabilized cells, as previously described (Baracca et al. 2013). Briefly, cells were incubated with 45 µg/ml digitonin in a Tris-HCl buffer (pH 7.4) and the synthesis of ATP driven by Complex I or Complex II was induced by adding either 10 mM glutamate/10 mM malate (+ malonate) or 20 mM succinate (+ rotenone), respectively. The reaction was started with 0.5 mM ADP and stopped 3 min later by adding dimethyl sulfoxide (80%) to the reaction mixture. Newly synthesized ATP was measured under basal condition by a bioluminescence assay kit based on the use of the luciferin/luciferase system, following the manufacturer’s instructions (Roche, Basel, Switzerland). The amount of ATP measured was normalized to the protein content.

### Flow cytometry analysis

Flow cytometry determination of ROS, the most contributors to total cellular reactive oxidant species, and superoxide anion was performed using a MUSE cytometer (Merk Millipore, Darmstadt, Germany) after loading the cells with either 5 µM CellROX Orange or 5 µM MitoSOX Red, respectively, as previously reported (Sgarbi et al. 2018b). Briefly, 24 h after seeding, the cells were incubated with each dye for 30 min at 37 °C, washed once with HBSS and then immediately trypsinized and processed. The cell fluorescence intensity was measured using a 532 nm excitation and a 576/28 nm emission filter; a total of 5,000 events were acquired for each analysis. Data analysis was performed by the Flowing software (Cell Imaging Core, Turku Centre for Biotechnology, University of Turku).

### Data analysis

All numerical data are expressed as mean ± SEM, as indicated. The number of biological replicates in independent experiments is detailed in each figure legend. The unpaired Student’s t-test, the One-sample t-test or the one-way analysis of variance (ANOVA) with Bonferroni’s post-hoc test was used, as indicated in the legend. Statistical analysis was performed by running GraphPad Prisme for Windows (GraphPad Software). A level of *P* < 0.05 was considered statistically significant.

## Results

### Warburg phenotype and IF_1_ in cancer cells

**Fig. 1:**
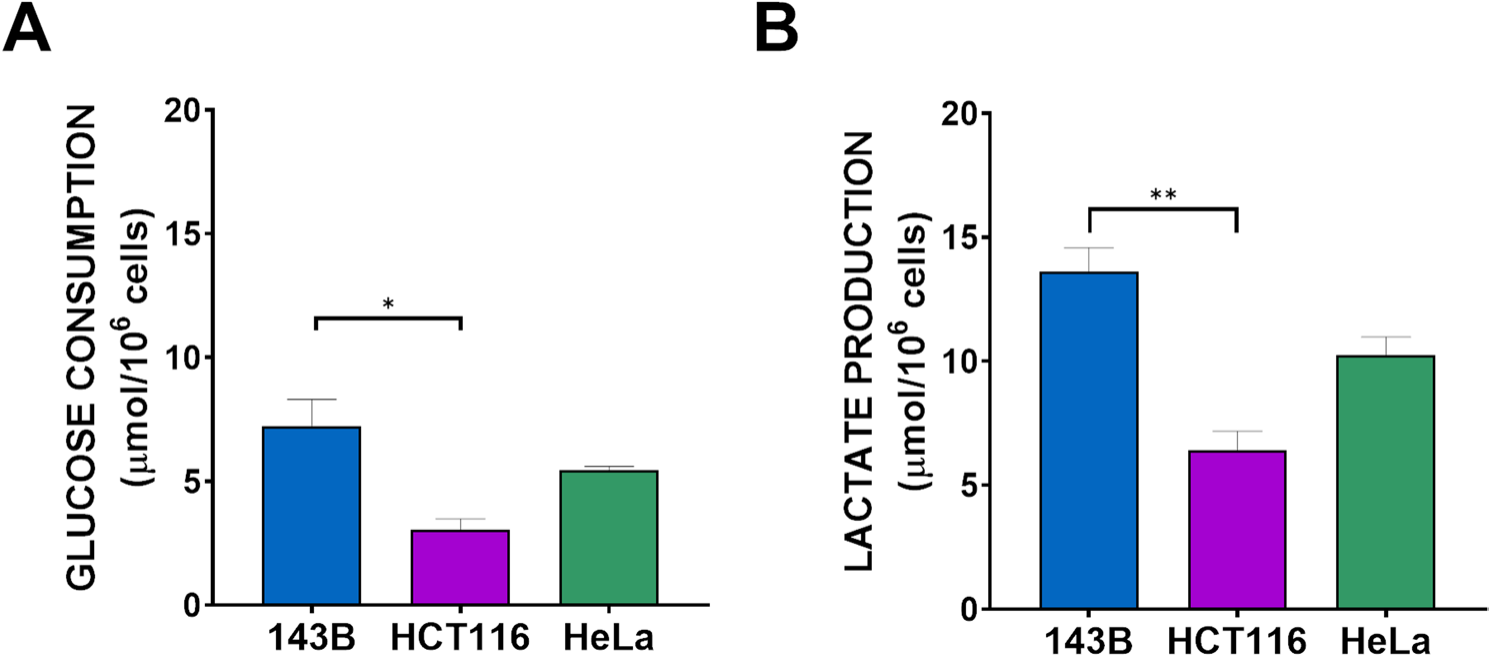
Glucose consumption and lactate production in parental cell lines. Metabolic parameters were analysed in 143B, HCT116, and HeLa cells after 24 h of growth (**A**,** B**). Values are means ± SEM. *p value < 0.05, **p value < 0.01, indicate statistical significance of 143B cell values compared to HCT116 and HeLa cells, as assessed by one-way ANOVA and Tukey’s test (*n* = 3 biological replicates)

**Fig. 2:**
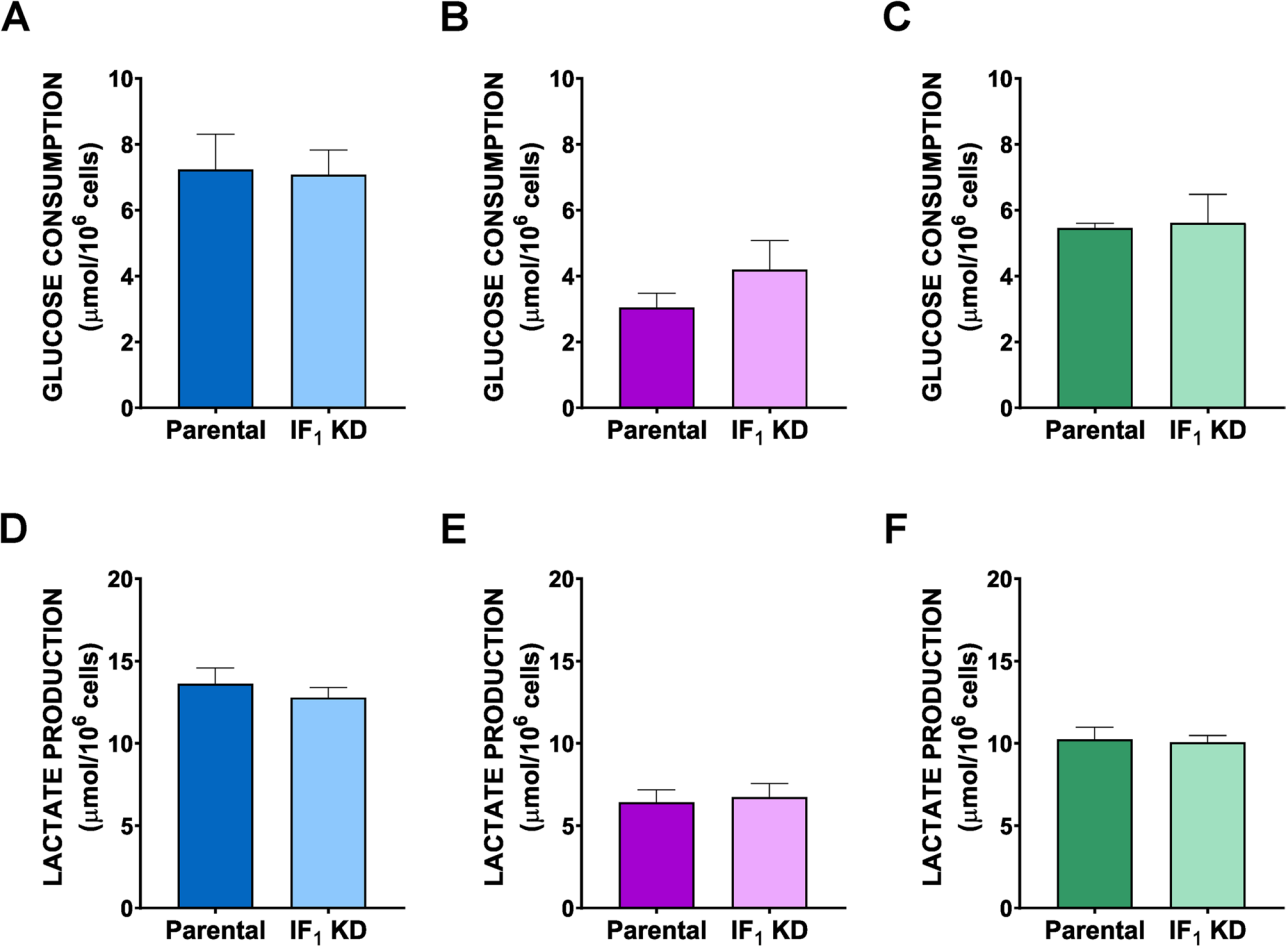
Glucose consumption and lactate release in parental cells and IF_1_-silenced derived clones. Metabolic parameters were analysed in 143B (**A**,** D**), HCT116 (**B**,** E**) and HeLa (**C**,** F**) cells after 24 h of growth. Values are means ± SEM. No statistical difference was detected between each parental cell line and the related IF_1_-silenced clone by Student’s t-test (*n* = 3 biological replicates)

The energy production of cells is primarily due to the catabolism of glucose and fatty acids. Glucose can be oxidized to pyruvate in the cytoplasm of cells and normally pyruvate enters into the mitochondria where it is further oxidized to CO_2_ and water. In many tumor cells pyruvate and NADH accumulate in the cytoplasm where they react together to produce lactate through a reaction catalyzed by lactate dehydrogenase (Le et al. 2010; Feng et al. 2018). Consumption of glucose in cancer cells can be particularly high since glucose in highly proliferating cells, besides supplying ATP, is used to produce the building blocks required for growth (amino acids, nucleotides, fatty acids) and the reducing potential in the form of NADPH. On this basis, to measure aerobic glycolytic flux it is necessary to evaluate glucose consumption, and other parameters that are closely related to the Warburg effect: lactate release and acidity of the cancer cell growth medium. The metabolic analysis was performed in three cancer cell lines (143B, HCT116, and HeLa), characterized by very different IF_1_ expression level as already reported (Sgarbi et al. 2024). Glucose consumption and lactate release were higher in 143B cells than in the other two cell lines, however only the difference between the values of 143B and HCT116 cells were statistically significant for both metabolites (Fig. 1). The stoichiometry ratio between lactate released and glucose consumed was about two for each tumor cell type, indicating that almost all glucose is glycolytically oxidized and then converted to lactate to produce energy (ATP). Notably, we found no statistically significant differences in either glucose consumption or lactate release between the parental cells and their derived IF_1_-silenced cells (Fig. 2). Incidentally, IF_1_ level in the three silenced clones was lower than 8% compared to the corresponding parental cells (Fig. S1).

**Fig. 3:**
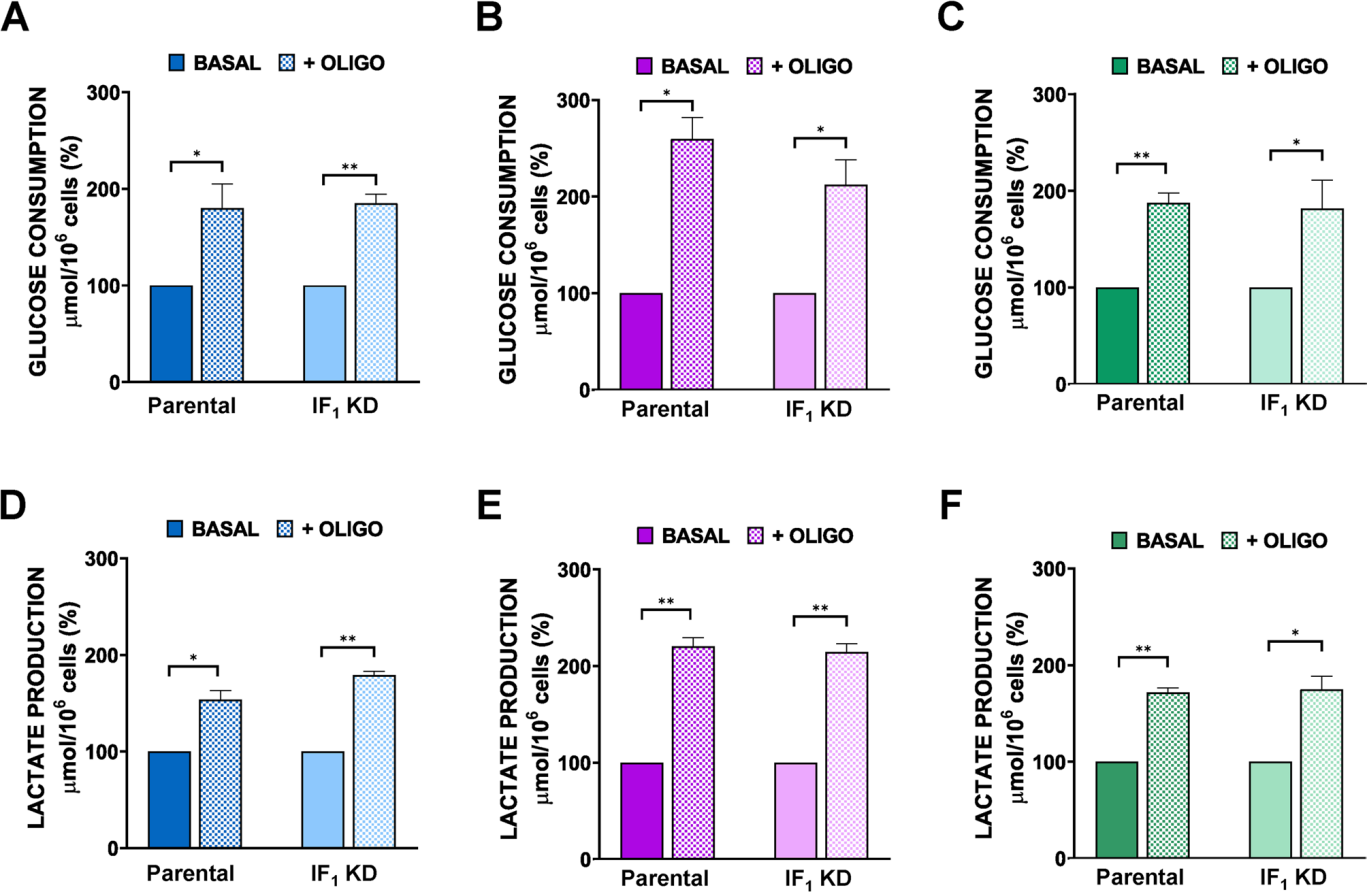
Glucose consumption and lactate release under basal conditions or in the presence of oligomycin. Metabolic parameters were analysed in 143B (A, D), HCT116 (B, E) and HeLa (C, F) cells after 24 h of growth. All oligomycin-related data were normalized to basal conditions. Values are means ± SEM. *p value < 0.05, **p value < 0.01 indicate statistical significance between the metabolic parameter values of each cell type grown under basal conditions or in the presence of 0.2 μM oligomycin, as assessed by Student’s t-test (*n* = 3 biological replicates). No statistical difference was detected between the values of each parental cells and the related IF_1_-silenced clone grown in the presence of oligomycin, as assessed by One-sample t-test (*n* = 3 biological replicates)

**Fig. 4:**
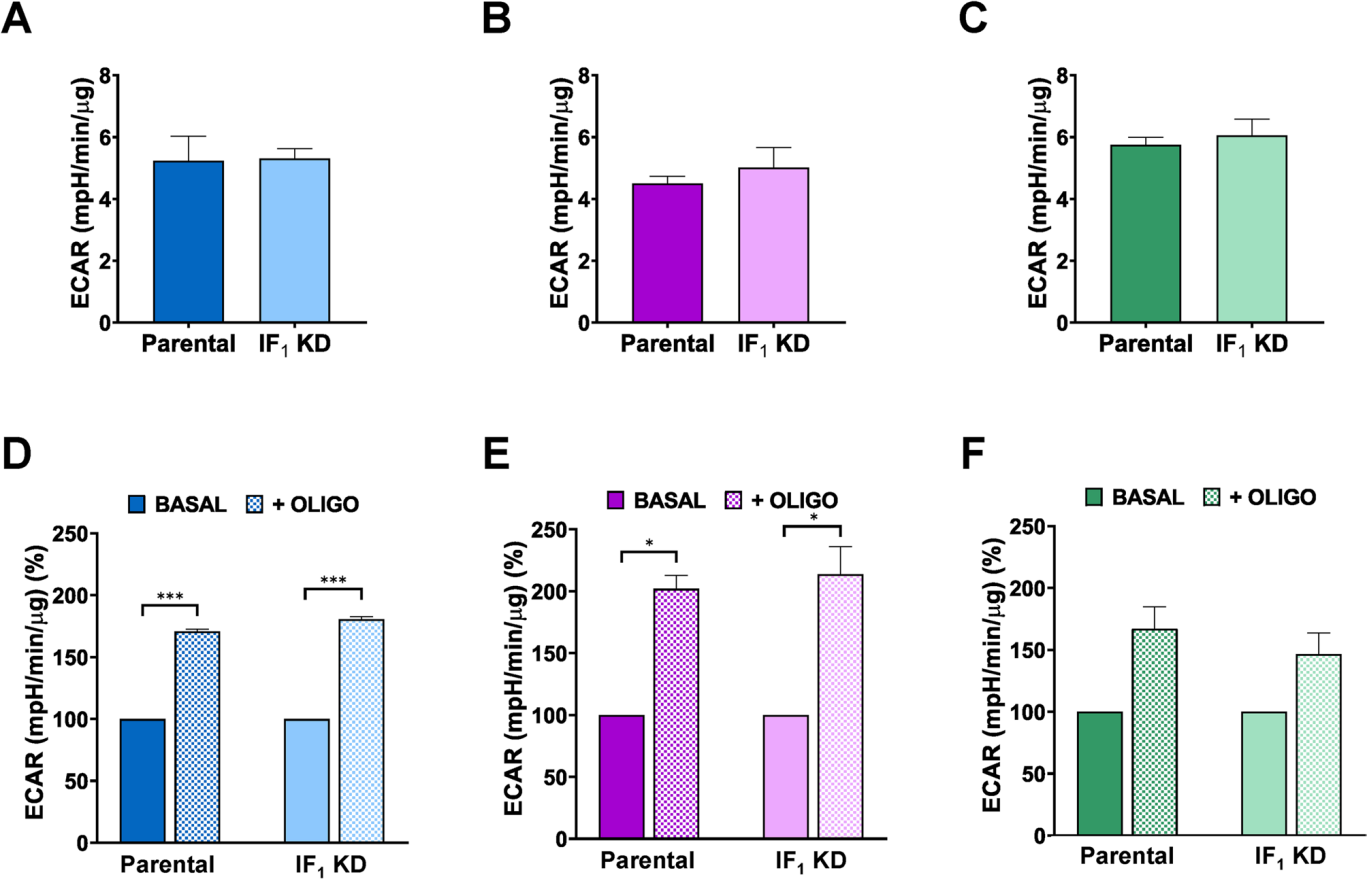
Extracellular acidification rate in parental cells and IF_1_-silenced derived clones. ECAR of all cell types was analyzed under basal conditions (**A**-**C**) and in the presence of 1 µM oligomycin (**D**-**F**). All values are normalized on the protein from cell lysates in each well and oligomycin-related data were normalized to basal conditions. Values are means ± SEM. *p value < 0.05, ***p value < 0.001 indicate the statistical significance, as assessed by Student’s t-test (**A**,** B**,** C**) or by One-Sample t-test (**D**,** E**,** F**) (*n* = 3 biological replicates)

Moreover, analyzing the effect of a low concentration of oligomycin (0.2 µM) on both metabolic parameters of all cell types, we observed a significant increase in glycolytic flux, as expected, that was qualitatively and quantitatively independent of the presence of IF_1_ (Fig. 3). Interestingly, oligomycin treatment of colon carcinoma cells resulted in a greater increase in glycolytic flux, as expressed by lactate release (Fig. S2), suggesting that colon carcinoma cells rely on OXPHOS to produce ATP more than other tumor cells tested. Furthermore, ECAR analysis performed by Seahorse under endogenous respiration conditions did not show any difference between parental and related IF_1_-silenced cells (Fig. 4A-C). Thus, good agreement was found between glycolytic flux as lactate released after 24 h of cell culture and ECAR in both parental and related IF_1_ KD cells. By treating cells with oligomycin (1 µM), ECAR similarly increased in culture media of parental and related IF_1_-silenced cells and again was higher in colon carcinoma cells (Fig. 4D-F).

**Fig. 5:**
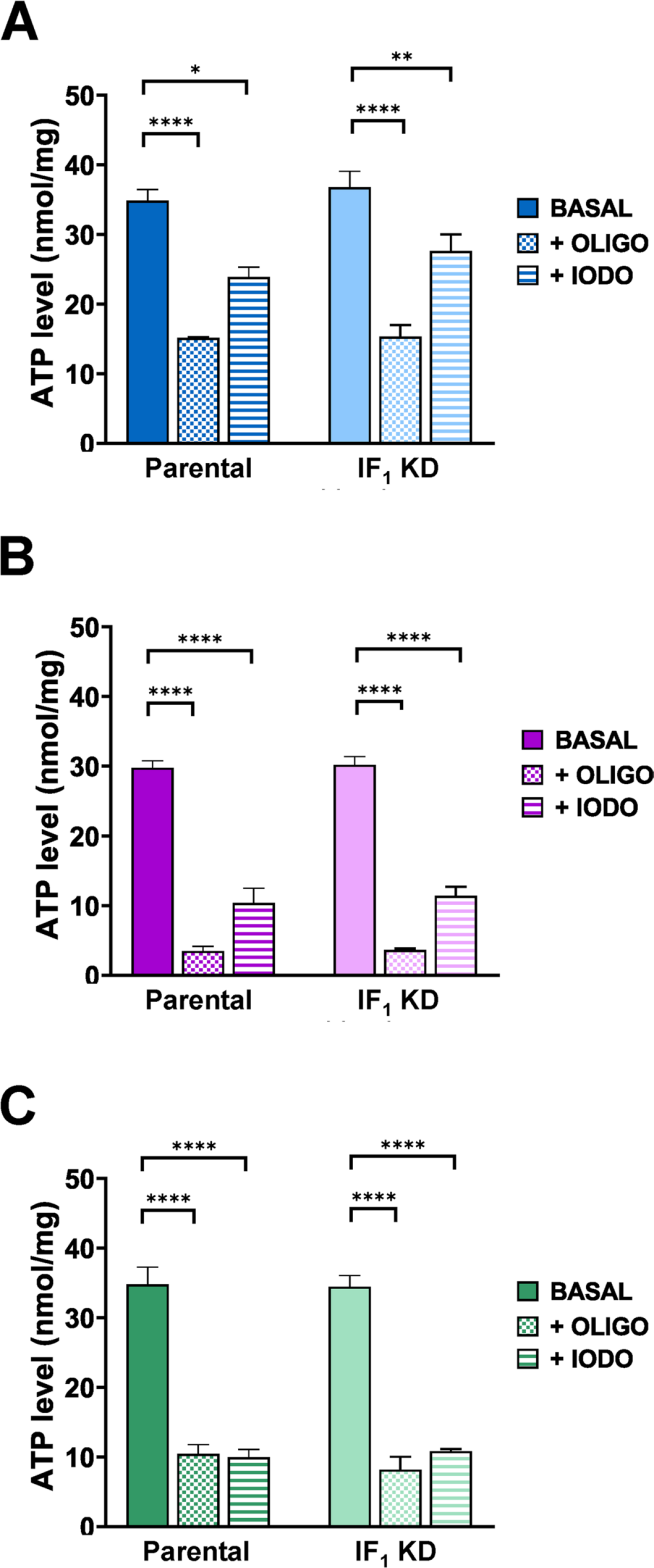
ATP levels of parental and IF_1_-silenced clones. ATP levels were evaluated in 143B (**A**), HCT116 (**B**) and Hela (**C**) parental cells and in their IF_1_-silenced clones under basal conditions (BASAL) and after treatment with 0.2 µM oligomycin (OLIGO) or 0.7 mM iodoacetamide (IODO) for 30 min. Values are means ± SEM. *p value < 0.05, **p value < 0.01, ****p value < 0.0001 indicate statistical significance of cellular ATP level values measured in the presence of inhibitors compared to basal conditions, as assessed by one-way ANOVA and Tukey’s test (*n* = 3 biological replicates)

A third interesting parameter that helps to characterize cancer metabolism is the assessment of the ATP level in cells when either glycolysis or OXPHOS is inhibited by iodoacetamide or oligomycin, respectively (Wolfson-Stofko et al. 2013; Zamaraeva et al. 2007). Figure 5 shows that ATP level of both parental and related IF_1_ KD cells treated with oligomycin ranged between 60 and 90%, indicating that all cell types significantly relied on OXPHOS to produce ATP. However, colon carcinoma cells showed the greatest sensitivity to oligomycin, whereas osteosarcoma cells showed the least responsiveness to the inhibitor. Intriguingly, ATP level of osteosarcoma cells was found scarcely inhibited (about 25–30%) by a low concentration of iodoacetamide (0.7 mM) compared to other tumor cell types (about 60–70%). However, the particularly high glycolytic flux estimated in this kind of tumor cells (Fig. 1) implies the presence of high levels of glucose transporters and glycolytic enzymes that can justify the modest effect of low iodoacetamide concentration on the ATP cellular level. Unlike other cell types tested, HeLa cells were similarly and remarkably sensitive to both inhibitors, suggesting that the energy metabolism of this cell type similarly relies on aerobic glycolysis and OXPHOS to produce ATP.

Interestingly, the results obtained from the above investigations on parental cell types and their IF_1_-silenced derived clones ruled out any role of the ATP synthase inhibitor protein in the modulation of energy metabolism and metabolic shift towards aerobic glycolysis (Warburg effect).

### Oxidative phosphorylation and IF_1_ in cancer cells

Mitochondrial bioenergetics plays a critical role in cancer cells not only as an energy supplier, but also because it can modulate the production of important precursors of molecules essential for cell growth, for homeostasis of ROS, and to facilitate resistance to apoptosis (Solaini et al. 2011; Moreno-Sánchez et al. 2023).

**Fig. 6:**
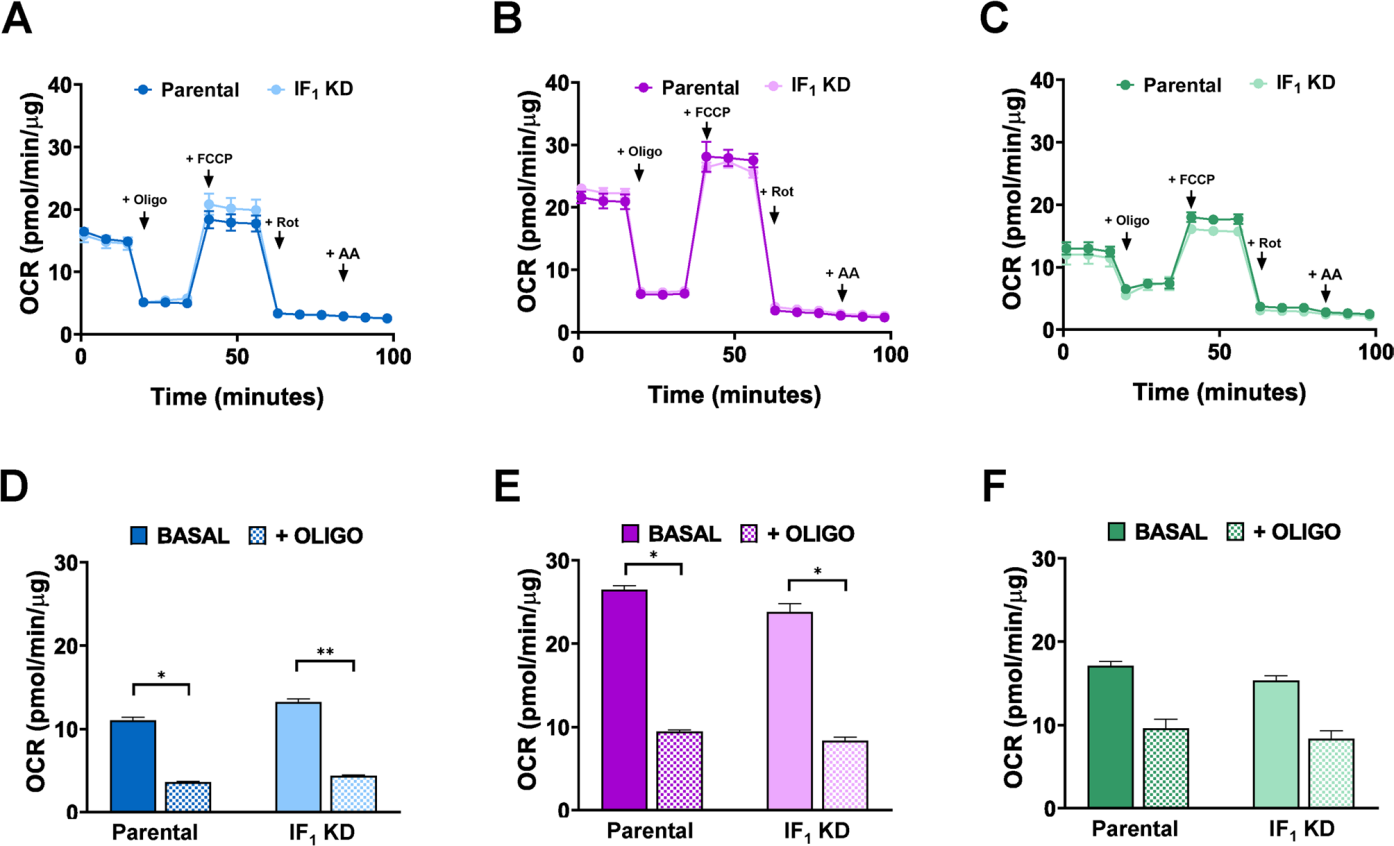
Oxygen consumption rate in parental and IF_1_-silenced cancer cells. Endogenous OCR traces of both parental cell lines (143B, HCT116, HeLa) and derived IF_1_-silenced clones **(A-C)**. OCR was measured under basal conditions and after the sequential addition of OXPHOS inhibitors (Oligo, oligomycin; Rot, rotenone; AA, antimycin) and uncoupler (FCCP), as detailed in Materials and methods. Histograms refer to basal and oligomycin inhibited OCR for both parental cells and their related IF_1_-silenced cells (**D-F**). All values are normalized to the protein from cell lysates in each well. (**A-C**) Values are means ± SEM; *p value < 0.05, **p value < 0.01 indicate statistical significance, as assessed by Student’s t-test (*n* = 3 biological replicates)

**Fig. 7:**
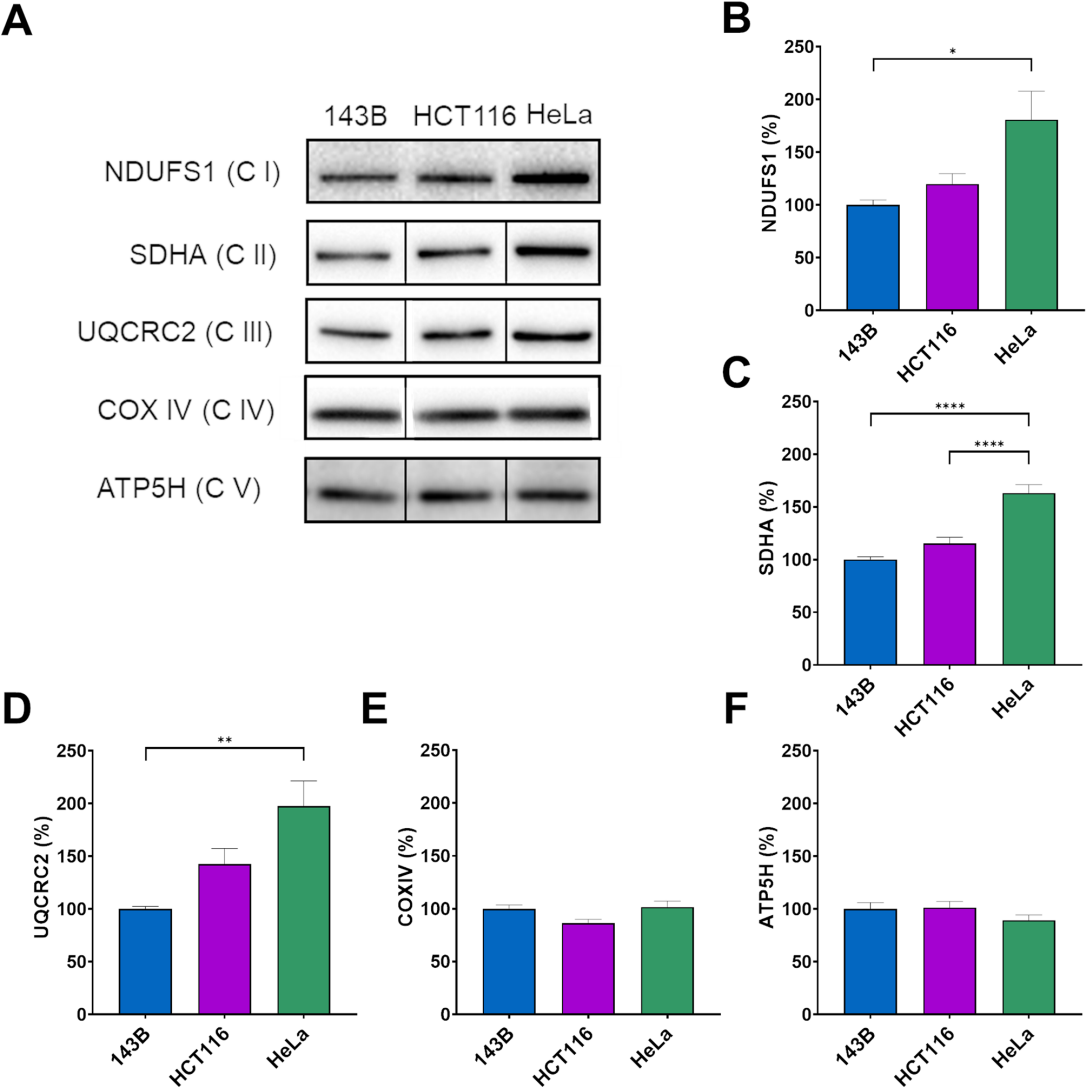
Levels of OXPHOS complexes in parental 143B, HCT116, HeLa cells. Representative immunodetection (**A**) and densitometric analysis of NDUFS1 (**B**), SDHA (**C**), UQCRC2 (**D**), COXIV (**E**), ATP5H (**F**) subunits of Complexes I, II, III, IV, and V, respectively, in parental 143B, HCT116 and HeLa cell lines. In the histograms, the values are reported as a percentage of the protein content normalized to that of 143B. Values are means ± SEM. *p value < 0.05, **p value < 0.01, ****p value < 0.0001 indicate the statistical significance, as assessed by ANOVA and Tukey’s test (*n* = 6–9 biological replicates)

We analyzed the respiration rate of our cancer cell lines and present OCR measured by Seahorse analysis (Fig. 6). The basal respiration rate was higher in colon carcinoma cells than in other tested tumor cells. The absence of IF_1_ did not affect endogenous respiration in any of the three types of tumor cells, consistent with the absence of any effect on their mitochondrial mass as previously reported (Sgarbi et al. 2024). Indeed, no difference in basal respiration nor in oligomycin-sensitive respiration (OSR) or in respiration uncoupled from ATP synthesis was found between silenced and IF_1_-expressing cancer cells (Fig. 6A-C). Quite surprisingly, the OCR of HeLa cells was found to be less sensitive to oligomycin than the other cancer cell types, osteosarcoma 143B and colon carcinoma HCT116 cells as highlighted in Fig. 6D-F. This observation is consistent with a rate of ATP synthesis (OXPHOS) in permeabilized HeLa cells similar to other cancer cells studied, despite the presence of a significantly higher mitochondrial mass as previously shown (Sgarbi et al. 2024). To shine light on this issue, we analyzed and quantified the OXPHOS Complexes in the three cancer cell lines, showing that the content of Complexes I to III was higher in HeLa cells than in the other cells, as expected, but Complexes IV and V (ATP synthase) were similar in all cell types (Fig. 7). This observation explains both the lower sensitivity to oligomycin and the similar activity of ATP synthase of HeLa cells compared to those of the other two cell lines (Sgarbi et al. 2024). The action of IF_1_ on the maximal rate of OXPHOS in digitonin-permeabilized cells energized by glutamate/malate and in presence of saturating ADP concentration is shown in Fig. S3. These data matched the results obtained on cellular respiration rate in intact cancer cells (OCR), again ruling out any role of IF_1_ as modulator of OXPHOS. Of notice, this conclusion agrees with observations reported by others (Fujikawa et al. 2012; Faccenda et al. 2017).

**Fig. 8:**
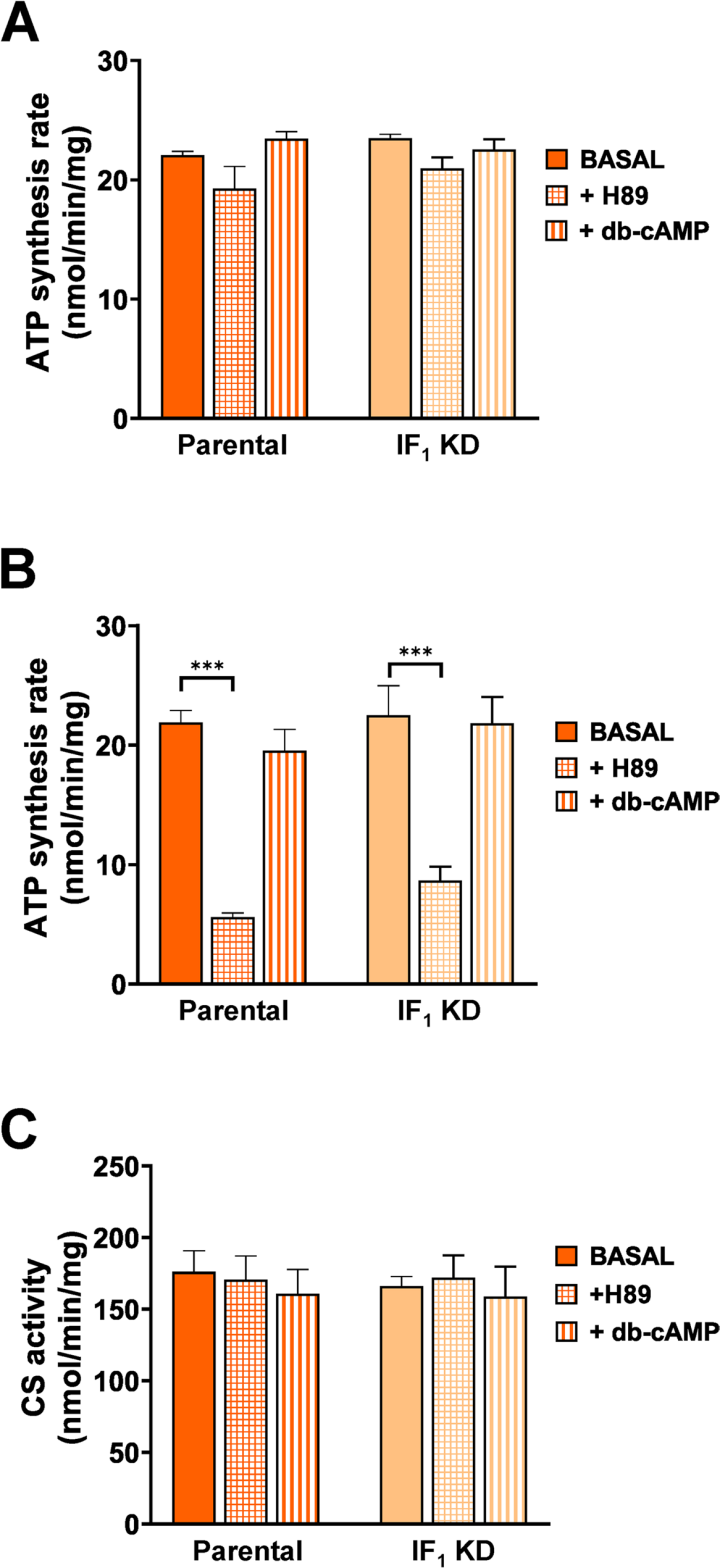
PKA effectors do not regulate ATP synthesis rate in HEK293 parental and IF_1_-silenced cells. Cells were exposed to the PKA effectors, H89 and db-cAMP, for 12 h before analysis. The rate of ATP synthesis driven by either succinate (**A**) or NAD-dependent substrates (**B**) was determined. Citrate synthase activity, as index of mitochondrial mass, was measured under basal conditions and after treatment with H89 or db-cAMP (**C**). Values are means ± SEM. ***p < 0.001 indicates statistical significance of values compared to basal condition using ANOVA and Tukey’s test (*n* = 3 biological replicates)

A still open issue is whether the inhibitory action of IF_1_ can be modulated by PKA-dependent phosphorylation (García-Bermúdez et al. 2015; Sgarbi et al. 2024; Carroll et al. 2024). We have shown that this does not occur in human cancer cells by maximizing and minimizing the possible phosphorylated and dephosphorylated state of IF_1_ (Sgarbi et al. 2024). However, to validate and extend our results, we addressed this issue in a human embryonic kidney cell line (HEK293) that expresses a very high level of IF_1_ (Sgarbi et al. 2018a). HEK293 is a transformed cell line, that is characterized by a high proliferative rate and behaves as a tumor line (Sgarbi et al. 2018a). Figure 8 shows that the ATP synthesis rate (OXPHOS) of parental and IF_1_-silenced cells again behave similarly whatever respiratory substrate was used to energize mitochondria. Moreover, the rate of ATP synthesis is independent on activation or inhibition of PKA activity by effectors such as dibutyryl-cAMP (db-cAMP) and N-[2-(p-bromocinnamylamino)ethyl]−5-isoquinolinesulfonamide (H89), respectively, when mitochondria are energized by succinate (Fig. 8A). Note that H89 treatment caused a significant decrease in the rate of ATP synthesis driven by NAD-dependent substrates (Fig. 8B) and neither effectors affected mitochondrial mass, as shown by CS activity analysis (Fig. 8C). All together these data exclude that other OXPHOS Complexes, but Complex I, were affected by PKA activity. Therefore, PKA-dependent phosphorylation of Complex I positively modulates OXPHOS activity while it has no effect on IF_1_ action, ruling out that the postulated inhibitory action of IF_1_ on the ATP synthesis activity of Complex V is modulated by its phosphorylated/dephosphorylated state. All the data match the results previously obtained in cancer cells and are consistent with the study recently published by Carroll an collaborators (Carroll et al. 2024), showing that human and bovine IF_1_ are potent inhibitors of ATP hydrolysis, with no effect on ATP synthesis and that IF_1_ action is not dependent on its possible phosphorylation.

### Metabolic reprogramming and ROS

**Fig. 9:**
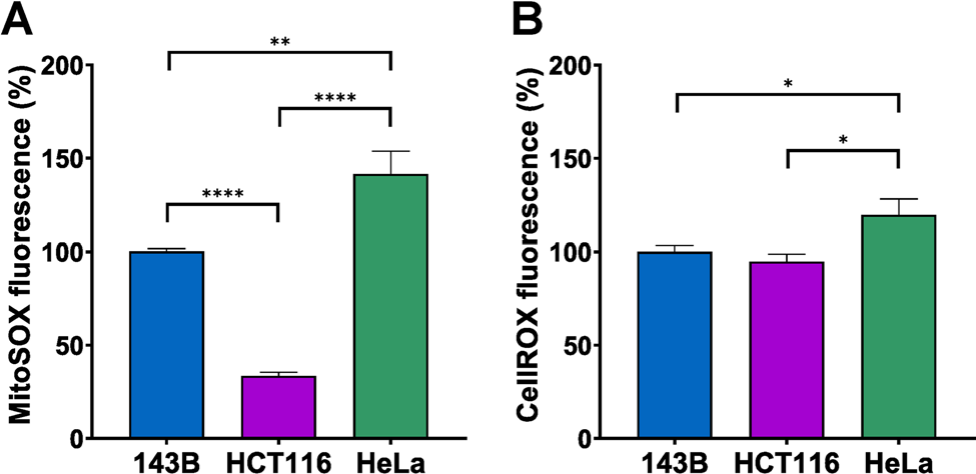
Reactive oxygen species in parental cell lines. Flow cytometry analysis of cells loaded with MitoSOX or CellROX was performed to assess the levels of superoxide anion (**A**) and ROS (**B**), respectively. In the histograms, mean fluorescence intensity values are reported as a percentage of the 143B values. Values are means ± SEM. *p value < 0.05, **p value < 0.01, ****p value < 0.0001 indicate statistical significance, as assessed by ANOVA and Tukey’s test (*n* = 9 biological replicates)

**Fig. 10:**
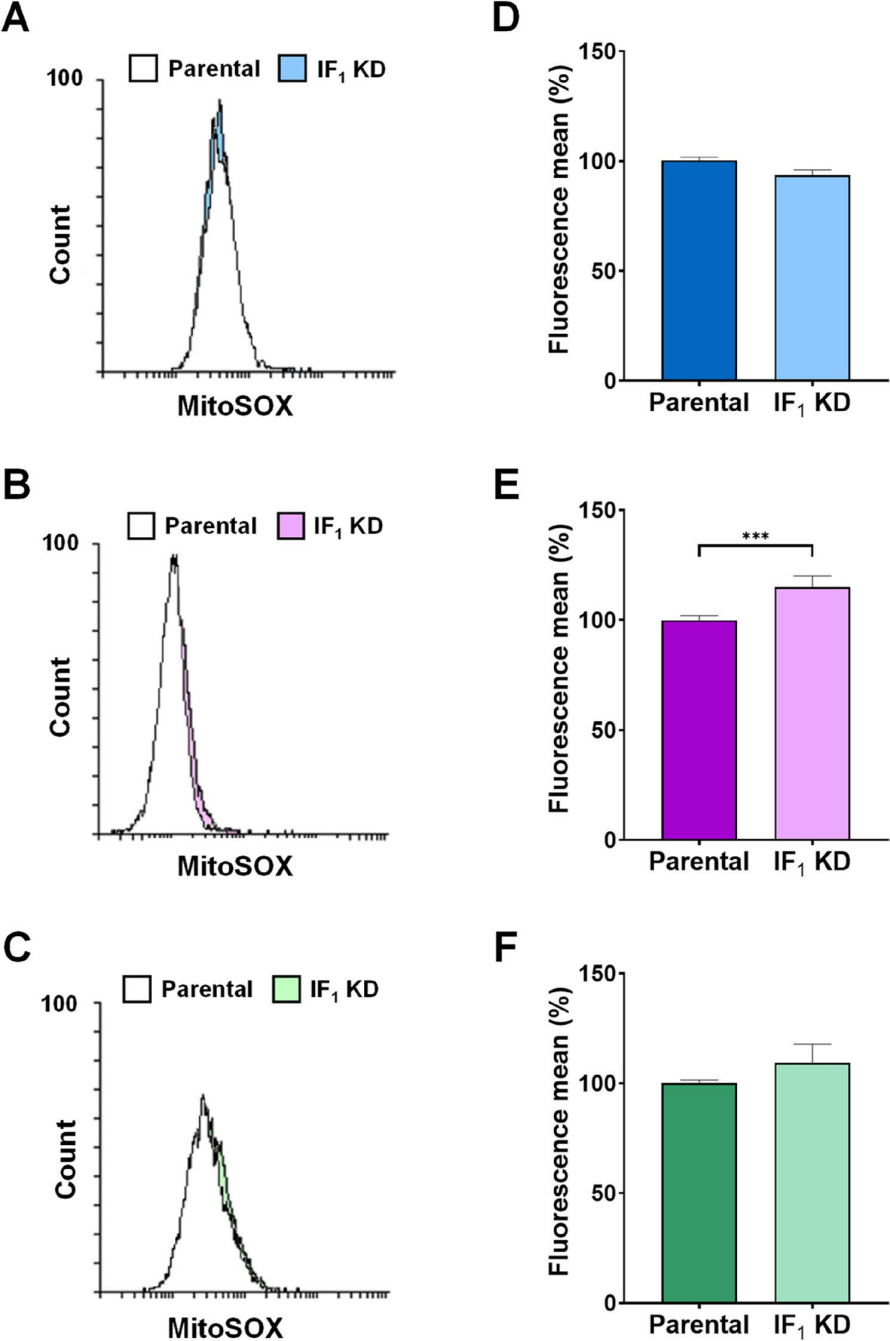
Superoxide anion level in parental and IF_1_-silenced cells. Representative flow cytometry analysis of MitoSOX-loaded 143B (**A**), HCT116 (**B**), HeLa (**C**) cells and their respective IF_1_-silenced clones. In the histograms, cellular levels of superoxide anion are expressed as mean fluorescence intensity values and those of IF_1_ KD clones are reported as a percentage of their respective parental cells (**D-F**). Values are reported as percentages ± SEM. ***p value < 0.001 indicates statistical significance, as assessed by Student’s t-test (*n* = 9 independent replicates)

**Fig. 11:**
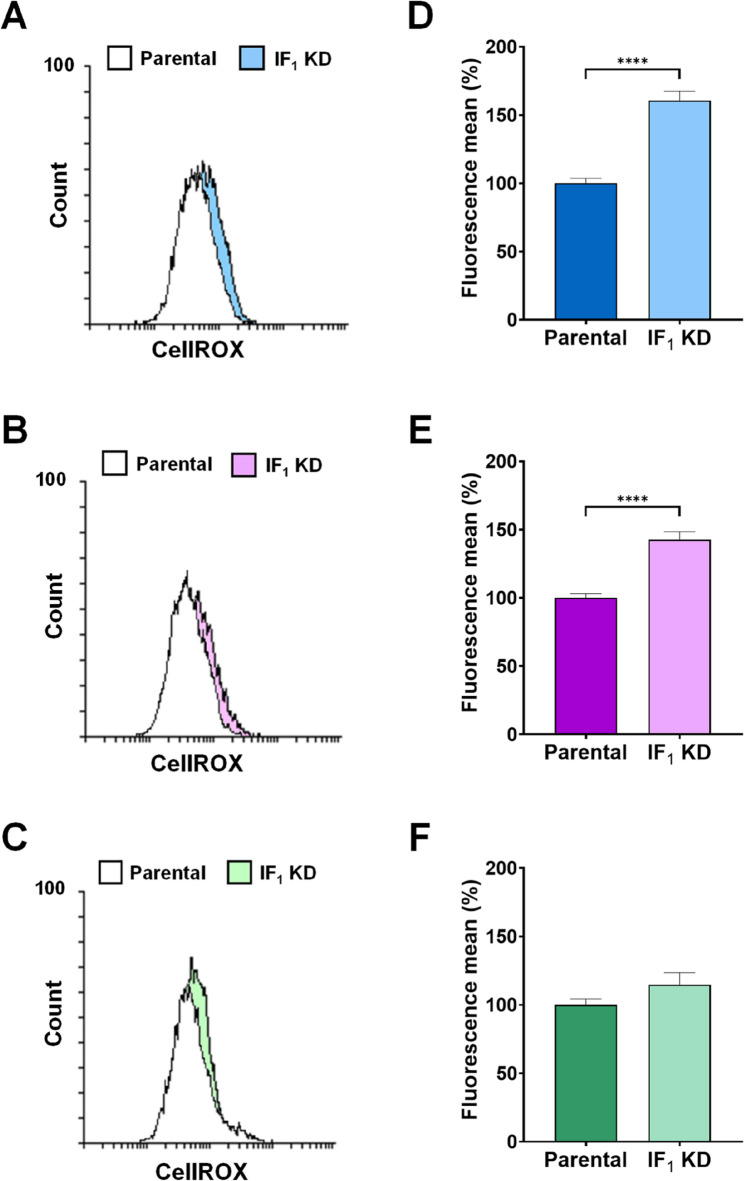
ROS level in parental and IF_1_-silenced cells. Representative flow cytometry analysis of CellROX-loaded 143B (**A**), HCT116 (**B**) or HeLa (**C**) cells and their respective IF_1_-silenced clones. In the histograms, cellular levels of ROS are expressed as mean fluorescence intensity values and those of IF_1_-silenced clones are reported as a percentage of their respective parental cells (**D-F**). Values are reported as percentages ± SEM. ****p value < 0.0001 indicates statistical significance, as assessed by Student’s t-test (*n* = 9 independent replicates)

Considering the relevant role played by ROS in modulating signaling pathways that favor growth and aggressiveness of cancers (Nakamura and Takada 2021; Cheung and Vousden 2022) and the still debated role of IF_1_ in promoting or inhibiting ROS production (Formentini et al. 2012; Sgarbi et al. 2018b), we choose to extend the investigation on ROS levels in all our cell models. Indeed, IF_1_ has been reported as a pro-oncogenic factor being able to enhance ROS generation by inhibiting the ATP synthase activity (Formentini et al. 2012). To assess the levels of superoxide anion and total ROS, all cell types were loaded with the fluorescent probes Mitosox and CellRox orange, respectively. Among the parental cell lines, HeLa cells exhibited the highest level of both superoxide anion and total ROS, both statistically significant compared to the other parental cells (Fig. 9). Moreover, comparison between parental and related IF_1_-silenced cells revealed that the distribution of Mitosox fluorescence of osteosarcoma or cervical carcinoma cells and their respective IF_1_ KD clones were similar (Fig. 10A, D and C, F). At variance, superoxide anion level was significantly lower in colon carcinoma cells than in related IF_1_-silenced clones (Fig. 10B, E). When the cells were loaded with CellRox orange to evaluate ROS levels, a significant increase in mean fluorescence was found in IF_1_ KD osteosarcoma and colon carcinoma cells, or a simple upward trend in IF_1_-silenced cervical carcinoma cells, compared to their related parental cells (Fig. 11). Overall, our results concur with previous observations made on osteosarcoma cells and exclude that IF_1_ overexpression may increase the cellular level of ROS favoring cancer cells survival and proliferation.

## Discussion

This work reports a set of typical parameters revealing the Warburg effect in cells from three different tumors, characterized by both different levels of IF_1_ expression and mitochondrial mass, and in their derived stable IF_1_ KD clones. Therefore, this study extends the investigation on the energy metabolism of cancer cells and completes the study on the possible role of IF_1_ in the metabolic reprogramming of cancer cells to the Warburg effect.

### Aerobic Glycolysis in cancer cells

Our results showed that most glucose consumed by all cancer cell types was converted into lactate and released to the culture medium: a clear-cut evidence of aerobic glycolysis, in accordance with a large body of experimental evidences (DeBerardinis et al. 2008; Semenza 2008; Potter et al. 2016; Jiang 2017; Moreno-Sánchez et al. 2023). As expected, the selective inhibitor of ATP synthase, oligomycin, similarly increased the lactate release and the acidification rate measured by Seahorse analysis in each type of parental and IF_1_ KD cells. Notably, colon carcinoma cells showed the lowest rate of glycolytic flux and the highest sensitivity of the analyzed metabolic parameters to oligomycin, suggesting that their energy metabolism is more dependent on OXPHOS. Having long experience in cell culture and being aware that almost all cultured cells, not just tumor cells, produce lactate when commonly grown in the presence of supraphysiological glucose and abundant oxygen (Liberti and Locasale 2016; Finley 2023), we also evaluated the contribution of OXPHOS to the ATP cellular level in parental cell lines derived from different tumor types to further enhance the knowledge of cancer bioenergetics. Indeed, our results concur with quite a few other researchers to sustain the important role of OXPHOS in cancer cell growth and proliferation since all cell types showed a high sensitivity to the ATP synthase inhibitor oligomycin that severely decreased their ATP level (−60 to −90% as shown in Fig. 5). Intriguingly, the ATP level of HeLa cells which are characterized by the highest mitochondria mass compared to osteosarcoma and colon carcinoma (Sgarbi et al. 2024), is not the most affected by oligomycin, as expected. However, this behavior of HeLa cells matches the increased glycolytic flux observed upon inhibition of OXPHOS by oligomycin (Fig. S2 and Fig. 3). One might speculate that such an unexpectedly high contribution of glycolysis to the bioenergetics of HeLa cells is due to the significantly lower enrichment of mitochondria in both Complex IV and ATP synthase compared to those of osteosarcoma and colon carcinoma cells (Fig. 7), and possibly to the presence of overexpressed mutated p53 (Moreno-Sánchez et al. 2023). Notably, the presence or absence of IF_1_ does not significantly affect any of the metabolic parameters analyzed in three different types of cancer cells, highlighting the absolute lack of influence of IF_1_ on the metabolic shift and acquisition of the Warburg phenotype in cancer cells.

### ATP synthase and IF_1_ in cancer cells

The F_1_F_o_-ATPase is a reversible enzyme that hydrolyzes ATP when Δµ_H_ + is collapsed, a condition occurring when cells are exposed to anoxia or near-anoxia conditions and the energy request is satisfied by the glycolytic flux (Al Tameemi et al. 2019; Solaini et al. 2021). Under these conditions IF_1_ binds to its canonical site located at the α_Ε_/β_Ε_ subunit interface and by blocking the enzyme activity limits the waste of ATP and promotes the survival of tumor cells (Solaini et al. 2021). Here we evaluated the role of IF_1_ in regulating ATP synthase when it synthesizes ATP through the Seahorse analysis. The results clearly demonstrated that there was no difference between basal, oligomycin-inhibited, and uncoupled respiration rates, regardless of whether IF_1_ was expressed or silenced in all cancer cell types examined (Fig. 6). Interestingly, endogenous respiration of colon carcinoma cells was found to be significantly higher than both osteosarcoma and cervix carcinoma cells. This observation is consistent with the lower glucose consumption/lactate production and the higher sensitivity of both metabolic parameters and cellular ATP level to oligomycin, confirming the higher dependence of HCT116 energy metabolism on OXPHOS. The results obtained by the Seahorse analysis on adherent cells expressing or not IF_1_ match previous results provided both by the polarographic assay on cell suspensions, using a Clark-type oxygen electrode, and by the ATP synthesis rate (OXPHOS) assay on permeabilized cells, using a luciferin/luciferase chemiluminescence method (Sgarbi et al. 2024).

Once established that the examined tumor cell lines satisfy their energy requirements for growth through a combination of aerobic glycolysis and OXPHOS, we would like to mention that quantitative contribution of the two processes could be assessed (Mookerjee et al. 2017). Although we did not apply the rather rigorous method reported in the above mentioned paper, we could have a clear-cut evidence of a prevalent consumption of glucose to produce lactate in all cancer cells examined by comparing the histograms of Figs. 2 and 3. The contribution of OXPHOS to ATP supply, although relevant in all cells, was highest in HCT116 colon carcinoma which showed the highest OCR under basal conditions and a marked sensitivity of energy metabolism to oligomycin (Fig. 6 and S2), which was associated with the greatest increase of extracellular acidity compared to 143B and HeLa cells (Fig. 4). The present work also provides further evidence that PKA does not regulate the action of IF_1_ and does not modulate its ability to inhibit the physiological activity of ATP synthase. Indeed, as shown, it occurs in cells expressing high levels of IF_1_, regardless of their tumor or immortalized nature, as in the case of the HEK293 cell line (Fig. 8). Consequently, taken together, the results obtained in both immortalized and cancer cells established that the possible PKA-dependent phosphorylation of IF_1_ does not change the issue that IF_1_ plays no role in the regulation of physiologically functioning ATP synthase and hence in OXPHOS (Sgarbi et al. 2024). However, OXPHOS rate was found significantly decreased when Complex I-dependent substrates were used to fuel mitochondria in cells pre-treated with PKA inhibitor, but this was expected since OXPHOS can be regulated by kinases-dependent phosphorylation of Complex I and Complex IV subunits (Papa et al. 1996; Chen et al. 2004; Acin-Perez et al. 2011). Accordingly, Carroll and colleagues (Carroll et al. 2024) demonstrated that phosphomimetic mutants of human IF_1_ were able to inhibit the ATP hydrolytic activity of bovine heart submitochondrial particles as does wild-type IF_1_. Thus, phosphorylation of IF_1_ does not prevent its ability to inhibit the hydrolytic activity of the ATP synthase Complex. Notably, the same authors did not observe any effect of wild-type human IF_1_ on the ATP synthesis rate (OXPHOS) of submitochondrial particles. Overall, data obtained in different experimental models concur in disputing the conclusions reported in a recent review (Cuezva and Domínguez-Zorita 2024). Finally, it is worth mentioning an intriguing paper proposing that IF1 regulates metabolic reprogramming involving its interaction with c-Myc and PGC-1α (Guo and Gu 2023), although our experiments in stable IF1 KD clones did not provide results different from the corresponding parental cells.

### ROS level in cancer cells and IF_1_

As we have shown, all tumor cell lines examined satisfy their energy requirements for growth through a combination of aerobic glycolysis and OXPHOS, both of which are associated with ROS production, but to different extents. Thus, considering the important roles of ROS in cellular metabolism, life, and death (Sainero-Alcolado et al. 2022), we also examined whether and to what extent mitochondrial and total reactive oxygen species were present in various cancer types and whether IF_1_ could influence the level of ROS.

The OXPHOS-rich HCT116 parental cells generated the lowest level of mitochondrial superoxide anion (Fig. 9A), and the level was significantly increased in the respective IF_1_-silenced clone (Fig. 10E). Nevertheless, total ROS level measured by CellRox was similar in HCT116 and 143B cells, although it was lower than that of HeLa cells (Fig. 9B). Interestingly, silencing of IF_1_ resulted in enhanced ROS levels in all tumor cell types, but this increase was statistically significant only in osteosarcoma 143B cells and colon carcinoma HCT116 cells (Fig. 11). Noteworthy, HeLa cells exhibited the highest levels of both superoxide anion and ROS (Fig. 9), an aspect that could be related to their low OXPHOS rate, taking into account their high mitochondrial mass. The reduced levels of ROS in parental cell lines compared to their IF_1_KD clones might be due to the ability of IF_1_ to promote mitochondrial homeostasis that protect cancer cells from death, by increasing the rate of both mitophagy and biogenesis, as we have shown in models of ischemia-reperfusion experiments (Righetti et al. 2023). Since mitochondrial quality control is cell type dependent (Malena et al. 2016), one might hypothesize that the higher efficiency of mitochondrial quality control mechanisms in IF_1_-expressing cancer cells is at the basis of both preservation of mitochondrial function and low ROS generation (Campanella et al. 2009; Faccenda et al. 2017). Note that under physiological conditions, the inhibitory action of IF_1_ could be exerted: (i) on some ATP synthases sensing low Δµ_H_ + in individual *cristae* within the same mitochondrion, which displays heterogeneous membrane potential (Wolf et al. 2019; Rieger et al. 2021), or (ii) during the assembly steps of the ATP synthase Complex (He et al. 2018), avoiding ATP waste and Δµ_H_ + enhancement. Moreover, IF_1_ expressed at high levels in cancer cells can interact with the OSCP subunit of ATP synthase and increase the resistance to apoptosis of cells by de-sensitizing the permeability transition pore (PTP) (Galber et al. 2023), a Ca^2+^-dependent high-conductance channel on the inner mitochondrial membrane (Petronilli et al. 1989). Considering that many cancer models inhibit cell death in response to fluctuations of the PTP effectors, such as Ca^2+^ or Mg^2+^, ROS, and matrix pH (Galber et al. 2020), we may hypothesize that IF_1_ inhibits the PTP-dependent apoptosis by a dual (direct and indirect) mechanism: (i) interacting with the ATP synthase and negatively modulating the PTP-opening and (ii) controlling and limiting cellular ROS level.

It is worth mentioning that it has long been observed that cancer cells have a higher production of ROS than normal cells, although it has since been recognized that tumorigenic signaling also increases the expression of antioxidant proteins that could counteract the elevated ROS production (Sullivan and Chandel 2014). Our results indicating lower ROS levels found in parental tumor cells compared to IF_1_ KD cells have been shared by several researchers (Campanella et al. 2009; Fujikawa et al. 2012; Wei et al. 2015; Sgarbi et al. 2018b; Zhong et al. 2023), but challenged by others (Formentini et al. 2012; Zhou et al. 2022). Indeed, in latter studies the authors found higher ROS levels in cells overexpressing IF_1_ compared to IF_1_ KD cells, but inconsistently a significant increase of ROS levels was also found in parental cells inhibited by oligomycin (García-Bermúdez et al. 2015). This result conflicts with ours, but we believe that it was provided by highly manipulated cellular models, far from those of pathological human cells that originally already expressed higher levels of IF_1_ than normal cells, that could give misleading results (see Supplementary Comments for details).

## Conclusions

In summary, in this study we provided a detailed analysis of bioenergetics in cells from three different tumor types. All the cells behaved similarly and most of the glucose consumption was converted into lactate, indicating that all the cells exhibited a typical Warburg phenotype. Noteworthy, all tumor cell types exhibited a different but significant contribution of OXPHOS to energy production. Furthermore, metabolic parameters were similar regardless of the presence or absence of IF_1_, demonstrating that IF_1_ overexpression does not represent a molecular mechanism taking part in metabolic rewiring of cancer cells (Warburg effect). Remarkably, the presence of IF_1_ was associated with a lower cellular level of ROS and this could contribute to the antiapoptotic role exerted by overexpressed IF_1_ in cancer cells. The present study conducted on 2D grown cells was necessary, but we would like to extend the investigation on the metabolic phenotype of tumor cells, in particular energy metabolism, using 3D-cell models, given the heterogeneity of cells in organoids, where the mitochondrial ATP production of cells is crucial to enable metastasis and resistance to anticancer drugs (Fiorillo et al. 2021).

## Supplementary Information

Supplementary Material 1.

## Data Availability

No datasets were generated or analysed during the current study.

## Abbreviations

OXPHOS: Oxidative phosphorylation
ECAR: Extracellular acidification rate
OCR: Oxygen consumption rate
KD: Knock-down
FBS: Fetal Bovine Serum
FCCP: Carbonylcyanide 4-(trifluoromethoxy) phenylhydrazone
ROS: Reactive oxygen species
db-cAMP: dibutyryl-cAMP
H89: N-[2-(p-bromocinnamylamino)ethyl]-5-isoquinolinesulfonamide
PTP: Permeability transition pore
