# Supplementary material for: Bioenergetics of cancer cells: insights into the Warburg effect and regulation of ATP synthase
Source: Mol Med. 2025 Oct 13;31:311. doi: 10.1186/s10020-025-01378-0 (PMC12519848; doi:10.1186/s10020-025-01378-0)
Supplement: Supplementary file 1 — Supplementary Material 1. [file 10020_2025_1378_MOESM1_ESM.docx]

**Supplementary Material**

**Supplementary Comments**

As a final comment, we believe that the appropriate selection of experimental approaches is a major challenge in evaluating biochemical and bioenergetic parameters*.* Indeed, to date, most of the available data on the inhibition of OXPHOS caused by IF_1_ in cancer cells to sustain its contribution to the Warburg effect are indirect and, in addition, obtained in cellular models where IF_1_ was overexpressed (Sánchez-Cenizo *et al*, 2010; García-Ledo *et al*, 2017; Zhou *et al*, 2022). Otherwise, all the work we have done on bioenergetics, mechanisms of adaptation to stress conditions, such as severe hypoxia, and resistance to cell death in cancer cells has been performed using and comparing parental cells and stably IF_1_ knocked down or knocked out cells (Sgarbi *et al*, 2018a, 2018b; Righetti *et al*, 2023; Galber *et al*, 2023; Sgarbi *et al*, 2024). We avoided to produce and investigate cells overexpressing IF_1_ for three main reasons: first, to study the bioenergetics of cells the use of models in which proteins are expressed in an uncontrolled manner is inappropriate, because this event consumes huge amounts of energy in cultured cells which could lead to an apparent decrease in the rate of OXPHOS assayed. Second, overexpression of a protein in cells may cause self-aggregation giving rise to the so called “amyloidosis” and IF_1_ is prone to aggregate, as shown by earlier works by Cabezon and colleagues (Cabezon *et al*, 2000). Amyloidosis can affect the cell biology and physiology, particularly damaging chemical and physical properties of membranes, including those of mitochondria (Aleardi *et al*, 2005; Willis & Patterson, 2013; Nor Ihsan *et al*, 2025), therefore, it may lead to draw misleading interpretation of the experiments. Third, it is reasonable to assert that excessive manipulated or heterogeneous biological systems as transiently transfected cells hardly provide good quality and easy to interpret data.

**Supplementary Figures**

**Fig. S1 Levels of IF_1_ in parental and IF_1_ KD clones.** Representative immunodetection of IF_1_ and β-actin (**A**) and densitometric analysis of IF_1_ normalized to their respective β-actin in 143B (**B**), HCT116 (**C**), HeLa (**D**). In the histograms, values are reported as a percentage of the protein content normalized to parental values. Values are means ± SEM. *** = p value < 0.001; **** = p value < 0.0001 indicates the statistical significance, as assessed by One-sample t-test (n = 3 biological replicates).

**Fig. S2 Lactate release in parental cells grown in the presence of oligomycin.** Data were reported as the percentage increase in lactate released from each cell type grown in the presence of the inhibitor compared to basal conditions. Values are means ± SEM. *p value < 0.05, **p value < 0.01, indicate statistical significance of HCT116 cell values compared to 143B and HeLa cells, all grown in the presence of oligomycin, as assessed by one-way ANOVA and Tukey’s test (n = 3 biological replicates).

**Fig. S3 ATP synthesis rate in permeabilized parental and IF_1_-silenced cells.** ATP synthesis rate is measured after 48 h in high glucose for 143B (**A**), HCT116 (**B**), HeLa (**C**) cells energized with 10 mM glutamate/malate plus 1.8 mM malonate by adding 0.5 mM ADP. Values are means of 3 independent experiments and are reported as percentages ± SEM normalized to parental cells.

**Supplementary References**

Aleardi AM, Benard G, Augereau O, Malgat M, Talbot JC, Mazat JP, Letellier T, Dachary-Prigent J, Solaini GC & Rossignol R (2005) Gradual Alteration of Mitochondrial Structure and Function by β-Amyloids: Importance of Membrane Viscosity Changes, Energy Deprivation, Reactive Oxygen Species Production, and Cytochrome c Release. *J Bioenerg Biomembr* 37: 207–225

Cabezon E, Butler PJG, Runswick MJ & Walker JE (2000) Modulation of the Oligomerization State of the Bovine F1-ATPase Inhibitor Protein, IF1, by pH. *Journal of Biological Chemistry* 275: 25460–25464

Galber C, Fabbian S, Gatto C, Grandi M, Carissimi S, Acosta MJ, Sgarbi G, Tiso N, Argenton F, Solaini G, *et al* (2023) The mitochondrial inhibitor IF1 binds to the ATP synthase OSCP subunit and protects cancer cells from apoptosis. *Cell Death Dis* 14: 54

García-Ledo L, Nuevo-Tapioles C, Cuevas-Martín C, Martínez-Reyes I, Soldevilla B, González-Llorente L & Cuezva JM (2017) Overexpression of the ATPase Inhibitory Factor 1 Favors a Non-metastatic Phenotype in Breast Cancer. *Front Oncol* 7: 69

Nor Ihsan NSM, Abdul Sani SF, Looi LM, Pathmanathan D, Cheah PL, Chiew SF & Bradley DA (2025) Raman spectroscopic signatures of amyloid fibrils: Insights into structural and biochemical changes in human tissues. *Biophys Chem* 325: 107480

Righetti R, Grillini S, Del Dotto V, Costanzini A, Liuzzi F, Zanna C, Sgarbi G, Solaini G & Baracca A (2023) The Pro-Oncogenic Protein IF1 Promotes Proliferation of Anoxic Cancer Cells during Re-Oxygenation. *Int J Mol Sci* 24: 14624

Sánchez-Cenizo L, Formentini L, Aldea M, Ortega ÁD, García-Huerta P, Sánchez-Aragó M & Cuezva JM (2010) Up-regulation of the ATPase Inhibitory Factor 1 (IF1) of the Mitochondrial H+-ATP Synthase in Human Tumors Mediates the Metabolic Shift of Cancer Cells to a Warburg Phenotype. *Journal of Biological Chemistry* 285: 25308–25313

Sgarbi G, Barbato S, Costanzini A, Solaini G & Baracca A (2018a) The role of the ATPase inhibitor factor 1 (IF1) in cancer cells adaptation to hypoxia and anoxia. *Biochimica et Biophysica Acta (BBA) - Bioenergetics* 1859: 99–109

Sgarbi G, Gorini G, Liuzzi F, Solaini G & Baracca A (2018b) Hypoxia and IF1 Expression Promote ROS Decrease in Cancer Cells. *Cells* 7: 64

Sgarbi G, Righetti R, Del Dotto V, Grillini S, Giorgio V, Baracca A & Solaini G (2024) The pro-oncogenic protein IF1 does not contribute to the Warburg effect and is not regulated by PKA in cancer cells. *Biochim Biophys Acta Mol Basis Dis* 1870: 166879

Willis MS & Patterson C (2013) Proteotoxicity and Cardiac Dysfunction — Alzheimer’s Disease of the Heart? *N Engl J Med* 368: 455–464

Zhou B, Caudal A, Tang X, Chavez JD, McMillen TS, Keller A, Villet O, Zhao M, Liu Y, Ritterhoff J, *et al* (2022) Upregulation of mitochondrial ATPase inhibitory factor 1 (ATPIF1) mediates increased glycolysis in mouse hearts. *J Clin Invest* 132: e155333
